# A man with panda eyes after a fall

**DOI:** 10.1002/ccr3.6233

**Published:** 2022-09-08

**Authors:** Dana Mahdi, Lydia Salem Yosief, Umar Butt, Aaron Cassidy, Andrzej Luckiewicz, Adnan Ather Malik, Amman Malik, Neel Jain, M. Adam Ali

**Affiliations:** ^1^ King's College London Medical School London UK; ^2^ Glenfield Hospital Leicester England, UK; ^3^ Department of General Surgery George Eliot Hospital NHS Trust Nuneaton UK; ^4^ School of Medicine University of Birmingham London UK; ^5^ Royal Free London Hospital NHS Foundation Trust London UK

**Keywords:** basal skull fracture, Battle's sign, panda eyes, periorbital ecchymosis

## Abstract

Most commonly caused by trauma, basal skull fractures present with a range of clinical signs. These include periorbital ecchymosis, as seen in this case, as well as rhinorrhea, otorrhoea and post‐mastoid ecchymosis. Suspected cases must be managed with appropriate imaging and medical or surgical treatment as indicated.

## Graphical abstract

Periorbital ecchymosis, known as Panda or Raccoon eyes, is seen when blood pools in subcutaneous periorbital tissues. It is a classical physical sign of basal skull fractures but can be seen in other conditions. Operative management is indicated in cases of associated intracranial hemorrhage, infection, or persistent CSF leak.

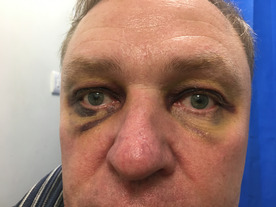

## 1 CASE PRESENTATION

**FIGURE 1:**
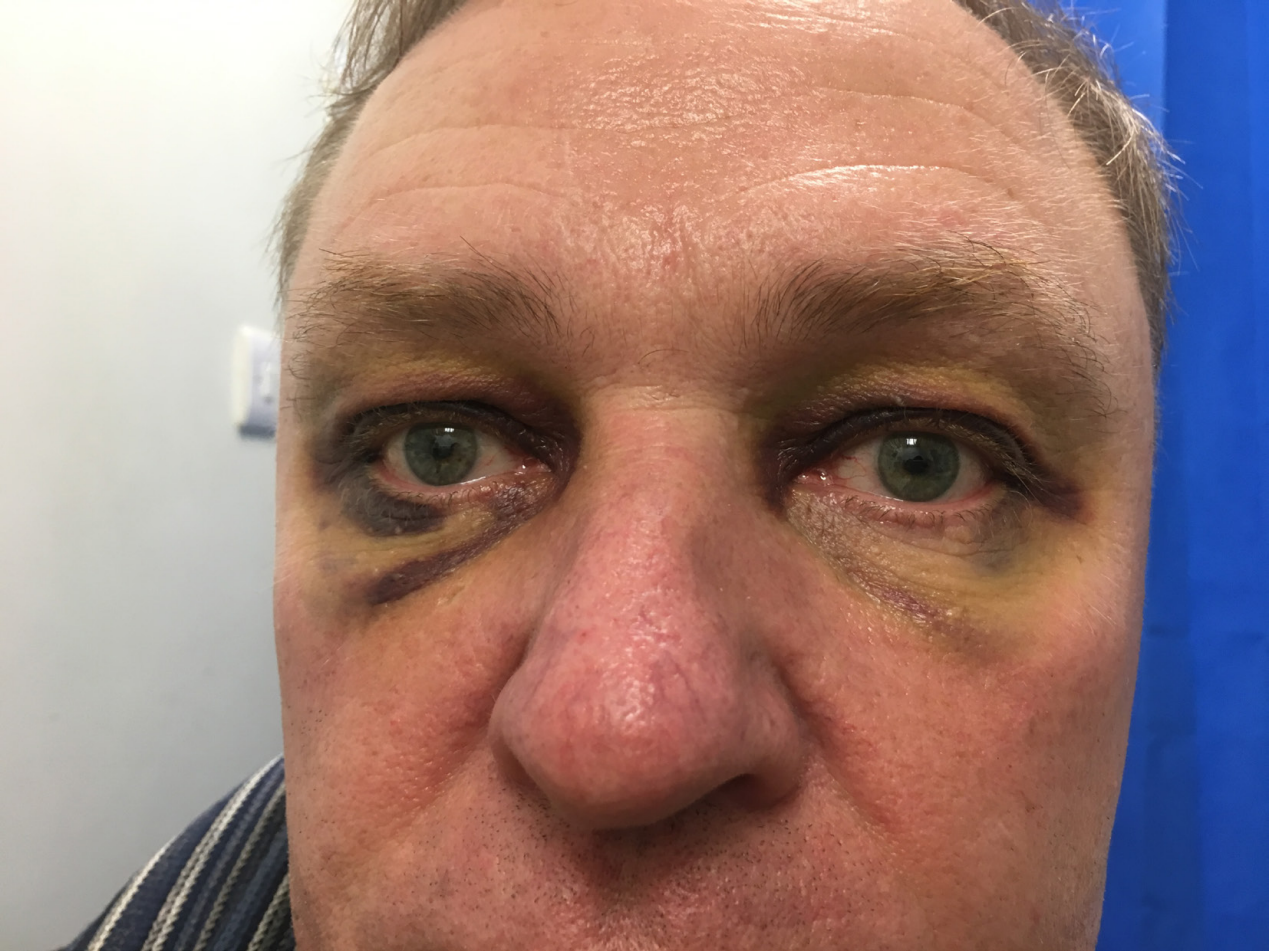
Photograph of periorbital ecchymosis

**FIGURE 2:**
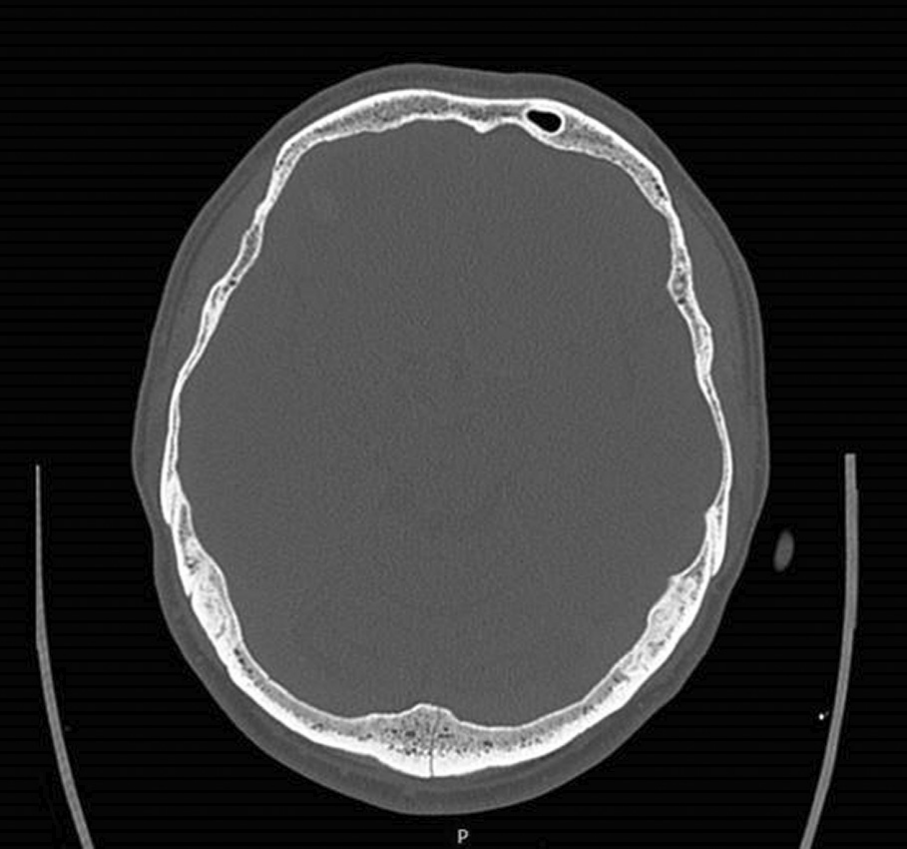
Computed tomography of the head, axial view

**FIGURE 3:**
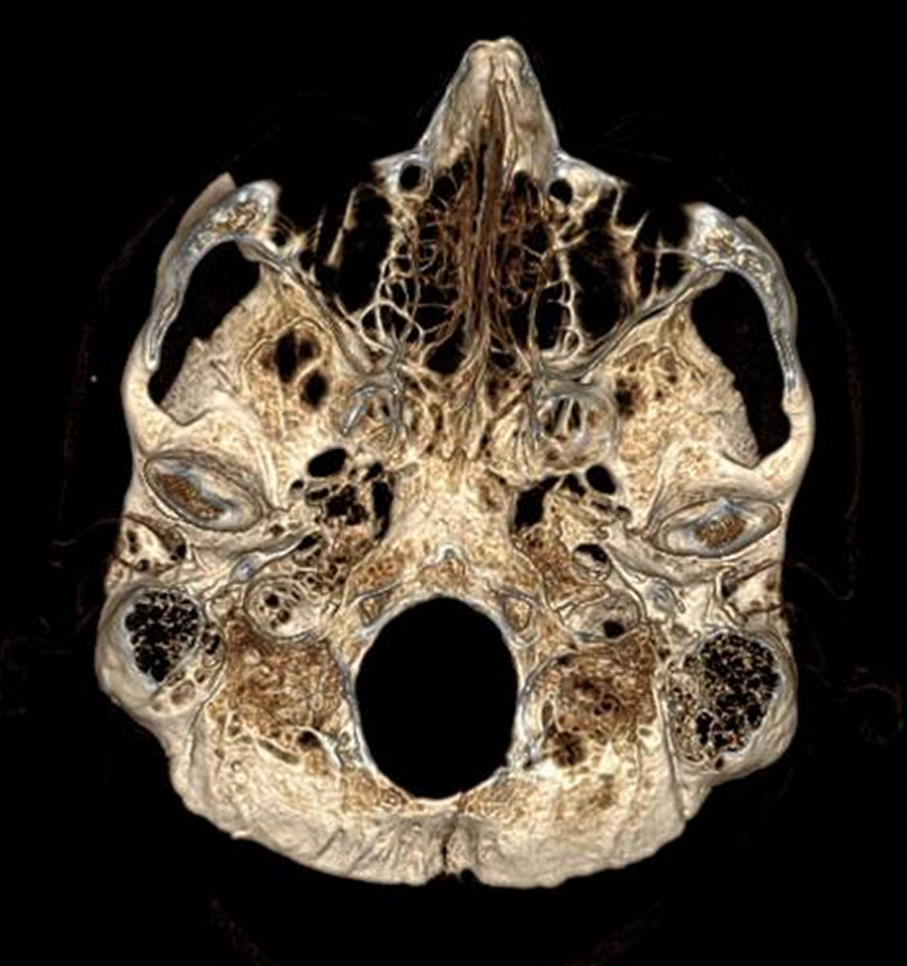
Three‐dimensional reconstruction showing an occipital fracture with involvement of the left transverse sinus

A 54‐year‐old man presented to the Emergency Department with bruising around the eyes (Figure 1). He had slipped on an ice‐covered surface and hit the back of his head 2 days earlier. His examination demonstrated periorbital ecchymosis, and his neurological examination was normal. Computed tomography of the head revealed a comminuted fracture of the occipital bone (Figure 2) involving the left transverse sinus and extending to the skull base (Figure 3).

## 2 DIAGNOSIS

Basal skull fracture with associated Panda eyes.

Periorbital ecchymosis, also known as Panda or Raccoon eyes, is a well‐recognized clinical sign of basal skull fracture. Other signs may include cerebrospinal fluid (CSF) oto‐ and rhinorrhea; post‐auricular mastoid ecchymosis (Battle's sign); hemotypanum and the Halo sign.^1^

Closed, non‐depressed skull fractures can be treated conservatively, if there is no evidence of intracranial pathology, neurological examination abnormality, or CSF leak. Surgical management is ordinarily pursued in cases of associated intracranial hemorrhage; persistent CSF leakage or gross wound contamination. This usually centers on debridement of devitalized tissues, evacuation of intracranial lesions, dural closure, and cranioplasty.^2^

Neurosurgical opinion was sought, and he was managed non‐operatively. Recommendations were made for further imaging, which the patient declined, and he was lost to follow‐up.

## AUTHOR CONTRIBUTIONS

MAA obtained the relevant radiologic images, consent from the patient and produced the manuscript. All authors worked collaboratively to finalise the manuscript, as well as make critical revisions of, and approve the final manuscript.

## CONFLICT OF INTEREST

None to declare.

## ETHICAL APPROVAL

None needed.

Signed consent obtained from the patient.

## CONSENT

Written informed consent was obtained from the patient to publish this report in accordance with the journal's patient consent policy.

## Data Availability

Data sharing not applicable to this article as no datasets were generated or analysed during the current study
